# Genome of *Linum usitatissimum* convar. *crepitans* expands the view on the section *Linum*

**DOI:** 10.3389/fgene.2023.1269837

**Published:** 2023-11-22

**Authors:** Ekaterina M. Dvorianinova, Elena N. Pushkova, Nadezhda L. Bolsheva, Elena V. Borkhert, Tatiana A. Rozhmina, Daiana A. Zhernova, Roman O. Novakovskiy, Anastasia A. Turba, Elizaveta A. Sigova, Nataliya V. Melnikova, Alexey A. Dmitriev

**Affiliations:** ^1^ Engelhardt Institute of Molecular Biology, Russian Academy of Sciences, Moscow, Russia; ^2^ Federal Research Center for Bast Fiber Crops, Torzhok, Russia; ^3^ Faculty of Biology, Lomonosov Moscow State University, Moscow, Russia; ^4^ Moscow Institute of Physics and Technology, Moscow, Russia

**Keywords:** flax, *Linum usitatissimum* convar. *crepitans*, section *Linum*, nanopore sequencing, *de novo* genome assembly

## Abstract

Sequencing whole plant genomes provides a solid foundation for applied and basic studies. Genome sequences of agricultural plants attract special attention, as they reveal information on the regulation of beneficial plant traits. Flax is a valuable crop cultivated for oil and fiber. Genome sequences of its representatives are rich sources of genetic information for the improvement of cultivated forms of the plant. In our work, we sequenced the first genome of flax with the dehiscence of capsules—*Linum usitatissimum* convar. *сrepitans* (Boenn.) Dumort—on the Oxford Nanopore Technologies (ONT) and Illumina platforms. We obtained 23 Gb of raw ONT data and 89 M of 150 + 150 paired-end Illumina reads and tested different tools for genome assembly and polishing. The genome assembly produced according to the Canu—Racon ×2—medaka—POLCA scheme had optimal contiguity and completeness: assembly length—412.6 Mb, N50—5.2 Mb, L50—28, and complete BUSCO—94.6% (64.0% duplicated, eudicots_odb10). The obtained high-quality genome assembly of *L. usitatissimum* convar. *crepitans* provides opportunities for further studies of evolution, domestication, and genome regulation in the section *Linum*.

## 1 Introduction

Plant genomes demonstrate high variability in size and content (Li et al., 2022; Sun et al., 2022). Genome sequences enable studying the beneficial features of agricultural plants and modifying and improving the desired traits (Nützmann et al., 2016; Sedeek et al., 2019; Wang et al., 2020; Choudhury et al., 2022; Singh et al., 2022; Tello-Ruiz et al., 2022). Therefore, plant genomics and pan-genomics open vast opportunities for breeding and agriculture. Knowledge of genome structure can unveil the mechanisms of regulation of key agricultural traits and highlight possible large-scale differences between the representatives of a taxon. In addition, plant genomes can spur studies on the evolution, domestication, and adaptation processes and the emergence of metabolic diversity driven by whole genome duplication (Song et al., 2021; El Karkouri et al., 2022; Petereit et al., 2022; Zhou and Liu, 2022; Bartlett et al., 2023).

*Linum usitatissimum* L. is a dual-purpose agricultural plant providing two main raw products of multipurpose use—seed and fiber (Nag et al., 2015). Flax seed is a source of biologically active compounds beneficial for human health (Kezimana et al., 2018; Sirotkin, 2023). Flax seed in animal feed also causes positive effects on immunity and growth (Salem et al., 2023). Flax oil of a certain fatty acid composition is actively used in the coating industry (Wang and Padua, 2005; Dmitriev et al., 2020a). In addition to the use in textile production (Van der Werf and Turunen, 2008), flax fiber serves as a component of composite materials (More, 2022). Flax biomass can be used as a source of bioenergy (Batog et al., 2023). Thus, *L. usitatissimum* is an important agricultural crop, and data on its diversity at the genome level can be implicated in breeding and understanding the evolution in the section *Linum*.

Genome sequences of flax representatives are useful sources of information for both basic and applied studies. Currently, seven *L. usitatissimum* assemblies are available in the databases (NCBI and Zenodo) (You et al., 2018; Dmitriev et al., 2020b; Zhang et al., 2020; Sa et al., 2021; Dvorianinova et al., 2022; Zhao et al., 2023). In the species of the section *Linum*, apart from *L. usitatissimum*, a genome of *Linum bienne* Mill. (considered to be a wild ancestor of cultivated flax) is available in the NCBI database (Zhang et al., 2020). Our study aimed at assembling a high-quality genome of *L. usitatissimum* convar. *crepitans* (Boenn.) Dumort. Convar. *crepitans* is a group of flax varieties with spontaneously opening capsules. It has been cultivated for fiber in Europe, but now it is not in use since seed shattering significantly complicates harvesting. However, it can be found in germplasm collections. The main feature of the convar. *crepitans* is the dehiscence of its capsules, but in other ways, it is quite similar to *L. usitatissimum* convar. *usitatissimum* (Muir and Westcott, 2003). The genetic resource of the convar. *crepitans* is limited (Diederichsen, 2019). Nevertheless, the investigation of this convar. can significantly broaden the data on genetic diversity and domestication of *L. usitatissimum*. The genome assembly of the convar. *crepitans* can be incorporated in pan-genomic studies of flax, including the construction of pan-genome, mining key agricultural traits, and establishing the evolution of flax forms.

## 2 Material and methods

### 2.1 Plant material

The seeds of *L*. *usitatissimum* convar. *crepitans* K-1531 were provided by the Institute for Flax (Torzhok, Russia). The seeds were sterilized in a 1% NaClO solution for 5 min and then germinated on Petri dishes. High-quality seedlings were transplanted into the soil and grown for 3–4 weeks. After that, the tops of the plant branches were covered with a dark cloth to prevent exposure to light for 1 week. This step was important to minimize the level of metabolites in flax leaves before DNA extraction. The leaves were collected, frozen in liquid nitrogen, and stored at −70°C until DNA isolation.

### 2.2 DNA extraction

Nucleus isolation and DNA extraction were performed according to the previously developed protocol (Dvorianinova et al., 2022). Additionally, part of the DNA was purified using the Circulomics Short Read Eliminator kit (SRE kit, Circulomics, United States). DNA concentration and quality were assessed using a Qubit fluorometer (Thermo Fisher Scientific, United States) and a NanoDrop spectrophotometer (Thermo Fisher Scientific), as well as by electrophoresis in 0.3% agarose gel.

### 2.3 Nanopore and Illumina sequencing

Three libraries were prepared for Nanopore sequencing according to the manufacturer’s protocol for SQK-LSK109 and SQK-LSK114 kits (Oxford Nanopore Technologies (ONT), United Kingdom). The first one was prepared from the SRE-purified DNA using the SQK-LSK109 kit (ONT) and sequenced on the FLO-MIN-106D R9.4.1 flow cell (ONT). The second one was prepared from the SRE-purified DNA using the SQK-LSK114 kit (ONT) and sequenced on the FLO-MIN-114 R10.4.1 flow cell (400 bps mode, ONT). The third one was prepared from the non-treated DNA using the SQK-LSK109 kit (ONT) and sequenced on the FLO-MIN-106D R9.4.1 flow cell (ONT).

An Illumina DNA library was prepared using the NEBNext Ultra II DNA Library Prep Kit for Illumina (New England BioLabs, United Kingdom) according to the manufacturer’s protocol. Sequencing was performed on a NovaSeq 6000 (Illumina, United States) instrument with a read length of 150 + 150 bp.

### 2.4 Data analysis

Raw FAST5 sequences were converted into FASTQ format by Guppy 6.4.6 using the super accuracy flip-flop algorithm (dna_r9.4.1_450bps_sup.cfg, dna_r10.4.1_e8.2_400bps_sup.cfg) and quality filtering (--min_qscore 8). The adapter sequences were removed with Porechop (https://github.com/rrwick/Porechop).

Draft genomes were assembled using Canu 2.2 (Koren et al., 2017) (set parameter: genome size = 400 m), Flye 2.9 (Kolmogorov et al., 2019) (set parameters: “--genome size 400 m,” “--nano-raw”), GoldRush (Wong et al., 2023) (set parameter: G = 4e6), and NECAT (Chen et al., 2021). Assembly quality was evaluated by the QUAST parameters (QUAST 5.0.2) (Gurevich et al., 2013) and the presence of universal single-copy orthologs (BUSCO 4.1.2, eudicots_odb10) (Simão et al., 2015). For the reference-based QUAST assessment, we used the first version of the genome and annotation of the *L. usitatissimum* variety CDC Bethune (https://ftp.ncbi.nlm.nih.gov/genomes/genbank/plant/Linum_usitatissimum/all_assembly_versions/GCA_000224295.1_LinUsi_v1.1/, GCA_000224295.1_LinUsi_v1.1_genomic.fna.gz, GCA_000224295.1_LinUsi_v1.1_genomic.gff.gz, and GCA_000224295.1).

To improve the quality of the draft assembly of the convar. *crepitans* genome, polishing was performed using ONT reads: Racon 1.5.0 (Vaser et al., 2017) (polishing with both R9.4.1 and R10.4.1 reads) and medaka 1.8.0 (https://github.com/nanoporetech/medaka) (-m r1041_e82_400bps_fast_g615; polishing with R10.4.1 reads). Illumina reads were trimmed (trailing:30) and filtered (minlen:50) using Trimmomatic 0.38 (Bolger et al., 2014) and then used for final polishing by POLCA from MaSuRCA 4.0.1 (Zimin et al., 2013).

To align Illumina reads to the final genome assembly of the convar. *crepitans*, BWA-MEM (Li, 2013) was used. To calculate the coverage percentage of the final genome assembly with Illumina reads, SAMtools depth (Li et al., 2009) (set parameters: -q0 -Q0) was run on the generated bam file, and the number of covered positions was calculated with the “wc -l” bash command.

Repeat content of *L. usitatissimum* genome assemblies was calculated with LTR_retriever 2.9.0 (Ou and Jiang, 2018), which includes the BuildDatabase (default parameters), RepeatModeler (“-engine ncbi”), and RepeatMasker (“consensi.fa.classified” file as input) modules.

## 3 Results

To assemble a high-quality genome of *L. usitatissimum* convar. *crepitans*, we performed whole-genome sequencing on the ONT and Illumina platforms. Three DNA libraries were prepared for ONT sequencing. In two DNA pools, short fragments were eliminated using the SRE kit (Circulomics, United States). For these DNA pools enriched with long fragments, we received 7.2 Gb (R9.4.1 flow cell) and 6.2 Gb (R10.4.1 flow cell) of raw ONT data with an N50 of 22.9 and 21.8 kb, respectively. For the library from the non-treated DNA, we received 9.6 Gb (R9.4.1 flow cell) of raw ONT data with an N50 of 17.3 kb. After basecalling and adapter trimming, a total of 15.2 Gb of ONT data with an N50 of 21.8 kb remained. Then, we assembled draft genomes using Canu and Flye, which performed best in our previous study (Dvorianinova et al., 2022), as well as GoldRush and NECAT, which were not tested by us earlier.

**FIGURE 1:**
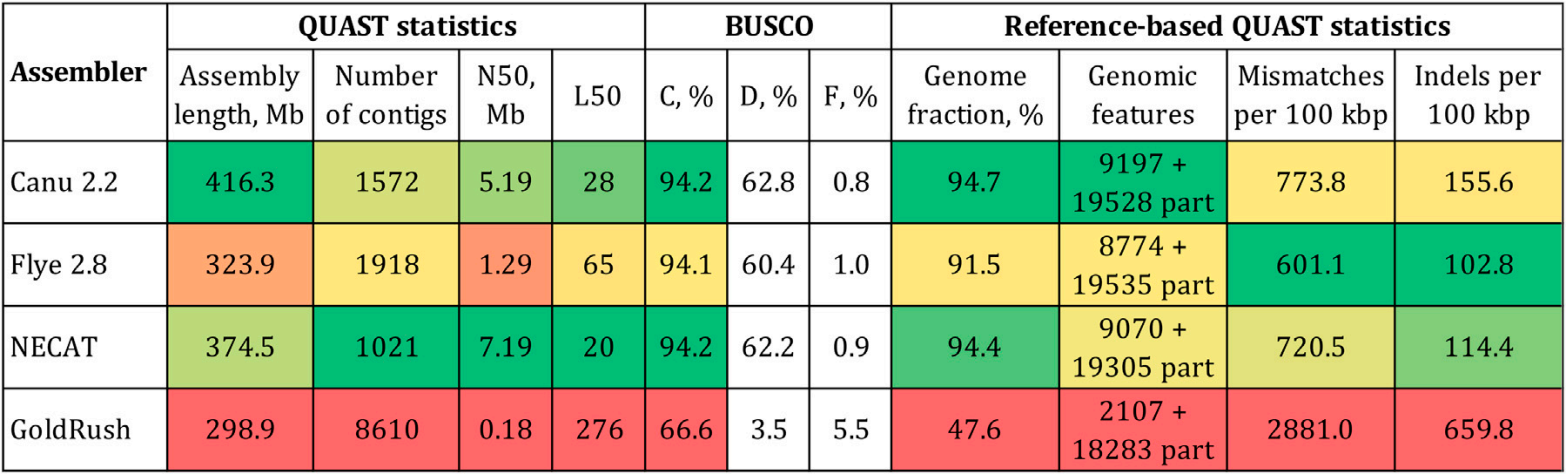
QUAST and BUSCO statistics for the *L. usitatissimum* convar. *crepitans* genome assemblies obtained with different tools. The green (best)–yellow–red (worst) color scale represents the quality of the values. BUSCO (eudicots_odb10): C—complete, D—duplicated, and F—fragmented. Genomic features: complete + partial; the detected feature from a reference genome is considered partial if it is covered by at least 100 bp.

The expected size of the *L. usitatissimum* genome was 400–450 Mb (You et al., 2018; Sa et al., 2021; Dvorianinova et al., 2022). Given the same size for the convar. *crepitans*, only Canu produced an assembly of a reasonable length—416.3 Mb (Figure 1). The assembly had an N50 of 5.2 Mb and the BUSCO completeness of 94.2% (eudicots_odb10). The assembly by NECAT had the same percentage of complete BUSCO and the highest N50 (7.2 Mb). However, the assembly length (374.5 Mb) was smaller than the expected one and might indicate the absence of important non-coding elements, e.g., repeats. Flye produced an assembly of an even smaller length—323.9 Mb. GoldRush demonstrated the worst performance among the tested software. The assembly was only 298.9 Mb long, had an N50 in the kb-range, and the BUSCO completeness of 66.6%.

The received draft assemblies were also assessed by the reference-based QUAST statistics (Figure 1). As a reference, we used the first version of the *L. usitatissimum* CDC Bethune genome (GCA_000224295.1) because it was assembled from accurate Illumina reads and annotated. Using a reference genome based on Illumina data is beneficial, as it contains errors different from those in a genome assembled from ONT data. In addition, the availability of annotation of the reference genome enabled us to calculate important QUAST statistics, e.g., the number of reference genomic features. The assembly by Canu had the highest fraction of the reference genome covered and the highest number of complete reference genomic features. The assembly by Flye had the lowest relative number of mismatches/indels. However, the accuracy of the obtained genome sequences can be improved by the polishing procedure. Therefore, we chose the assembly by Canu as optimal due to its length and the received parameters of contiguity and completeness.

**FIGURE 2:**
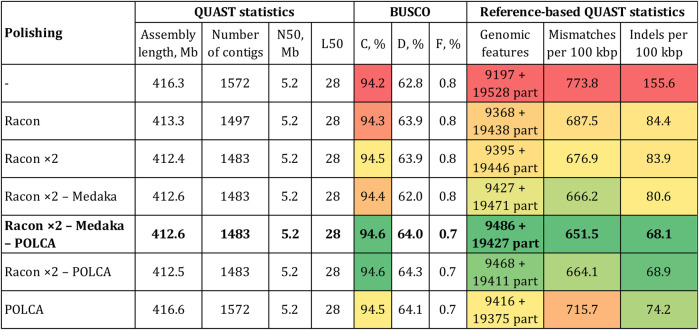
QUAST and BUSCO statistics for the Canu-assembled *L. usitatissimum* convar. *crepitans* genome polished with different tools. The green (best)–yellow–red (worst) color scale represents the quality of the values. BUSCO (eudicots_odb10): C—complete, D—duplicated, and F—fragmented. Genomic features: complete + partial; the detected feature from a reference genome is considered partial if it is covered by at least 100 bp.

Next, we improved the accuracy of the Canu-assembled sequences by polishing. To select polishers, we relied on the results of our previous studies. Two rounds of genome polishing with Racon and one round of polishing with medaka was the best combination for ONT reads (Dmitriev et al., 2020b; Krasnov et al., 2020; Melnikova et al., 2021; Dvorianinova et al., 2022). Therefore, we used this scheme for the genome of the convar. *crepitans* (Figure 2). Two iterations of Racon significantly decreased the relative number of mismatches and especially indels (by ∼2 times). The percentage of complete BUSCO and the number of complete reference genomic features increased. Polishing using medaka further improved the reference-based QUAST statistics. However, it slightly reduced the percentage of complete BUSCO (by 0.1%) and strongly reduced the percentage of duplicated BUSCO (by 1.9%). After all iterations of polishing with ONT reads (Racon ×2—medaka), the assembly length decreased by ∼4 Mb, compared to that of the draft assembly.

To improve the assembly accuracy to the maximum extent, we additionally polished the convar. *crepitans* genome (Canu—Racon ×2—medaka) with the generated Illumina data (89 M of 150 + 150 paired-end reads). According to our previous studies, POLCA was the most effective tool for Illumina reads (Dmitriev et al., 2020b; Krasnov et al., 2020; Melnikova et al., 2021). As a result of this procedure, the BUSCO completeness increased to 94.6% (by 0.2%), and the percentage of duplicated BUSCO increased to 64.0% (by 2.0%) (Figure 2). Thus, it eliminated the negative effect of medaka polishing, which caused the reduction in the parameter. Polishing with Illumina data also significantly increased assembly accuracy, according to the reference-based QUAST statistics.

In addition to polishing the draft Canu-assembled genome with both ONT and Illumina data, we tested whether it was possible to reach the same or better results using only Illumina reads or omitting the step of polishing by medaka. Thus, we polished the Canu and Canu—Racon ×2 assemblies with POLCA. The assembly polished using Racon (two iterations), medaka, and POLCA was more complete and accurate than the assemblies polished by Racon and POLCA or only POLCA (Figure 2). It had more complete reference genomic features and a lower relative number of mismatches and indels. However, the Canu–Racon ×2—medaka—POLCA assembly had a slightly lower percentage of duplicated BUSCO than the other two polished assemblies (Canu—Racon ×2—POLCA and Canu—POLCA), by 0.3% and 0.1%, respectively. Compared to genome assemblies polished using both ONT and Illumina reads, the assembly polished only using Illumina reads (Canu—POLCA) had significantly worse statistics of accuracy. Therefore, polishing with ONT reads could not be replaced with polishing only with short accurate reads.

Thus, the Canu—Racon ×2—medaka—POLCA scheme produced the most contiguous and complete assembly: length—412.6 Mb, N50—5.2 Mb, L50—28, and complete BUSCO—94.6%. Mapping Illumina reads to the convar. *crepitans* genome revealed that more than 398.5 million nucleotide positions were covered (96.6% of the sequence). According to SAMtools flagstat, 98.6% of the passed Illumina reads were mapped to the assembled genome. This indicated that the obtained genome assembly is of reasonable length and high completeness.

To compare the assembly of *L. usitatissimum* convar. *crepitans* with the available *L. usitatissimum* and *L. bienne* assemblies, the genomes were downloaded from the NCBI and Zenodo databases: 3896 (GCA_030674075.1), Atlant (GCA_014858635.1), Neiya No. 9 (https://zenodo.org/record/7811972), YY5 v.2 (https://zenodo.org/record/4872894), CDC Bethune v.1 and v.2 (GCA_000224295.1, GCA_000224295.2), Heiya 14 (GCA_010665265.1), Longya 10 (GCA_010665275.2), and *L. bienne* 15003 (GCA_010665285.1). For the downloaded assemblies, QUAST statistics were taken from the NCBI and Zenodo assembly descriptions (for the contig level) or calculated. To calculate BUSCO statistics, the eudicots_odb10 dataset was used. Among the analyzed genomes, the assembly of the convar. *crepitans* had one of the highest N50 and was the second most complete genome (after the assembly of Neiya No. 9), according to BUSCO statistics (Table 1).

**TABLE 1:** QUAST and BUSCO (C—complete, D—duplicated, and F—fragmented; eudicots_odb10) statistics and repeat content for the obtained *L. usitatissimum* convar. *crepitans* assembly (marked bold) and *L. usitatissimum* and *L. bienne* genome assemblies available in databases.

Flax variety	Sequencing platform	Assembly length, Mb	N50 (contig), Mb	Number of contigs	BUSCO			Total interspersed repeats, %
					C, %	D, %	F, %	
3896	ONT	447.1	6.2	1,695	93.8	62.3	0.7	49.3
Atlant	ONT and Illumina	361.8	0.4	2,458	94.4	63.4	0.7	44.7
Neiya No. 9	PacBio HiFi and Illumina	473.6	0.9	6,099	94.8	72.4	1.2	54.8
YY5 v.2	PacBio HiFi and BGI	455.0	9.6	336	94.5	63.1	0.7	50.1
**convar. *crepitans* K-1531**	ONT and Illumina	**412.6**	**5.2**	**1,483**	**94.6**	**64.0**	**0.7**	**49.9**
CDC Bethune v.1	Illumina	282.2	0.02	48,397	93.9	60.4	1.3	33.3
CDC Bethune v.2	Illumina	316.2	0.02	24,829	93.7	57.4	0.9	27.7
Heiya 14	Illumina	303.7	0.3	4,581	94.5	62.6	0.9	36.1
Longya 10	Illumina	306.4	0.2	4,419	94.4	60.5	0.9	36.0
*L. bienne* 15003	Illumina	293.6	0.1	6,369	93.3	50.4	1.3	36.3

The obtained convar. *crepitans* assembly contained 49.9% of total interspersed repeats (Table 1) per ∼413 Mb (assembly length). Meanwhile, *L. usitatissimum* genome assemblies from long reads had 44.7%–54.8% of repetitive sequences per 362–474 Mb. Flax genome assemblies from short reads comprised only 27.7%–36.3% of total interspersed repeats and had a smaller size (294–316 Mb).

## 4 Discussion

Plant genomes became the foundation of studies on the regulation of genetic features and their involvement in metabolic pathways, species evolution, and adaptation. Currently, genome sequencing is routine but relevant for agricultural plants. The genomes of crops are indispensable for modern breeding based on molecular procedures and targeted improvement of valuable plant features (Dmitriev et al., 2022). Furthermore, the availability of several diverse genome sequences for a species is key to the discovery of novel useful agricultural traits. For *L. usitatissimum*, seven genome sequences of different varieties were received earlier (You et al., 2018; Dmitriev et al., 2020b; Zhang et al., 2020; Sa et al., 2021; Dvorianinova et al., 2022; Zhao et al., 2023). In this work, we sequenced the genome of *L. usitatissimum* convar. *crepitans* which is no longer cultivated due to the dehiscence of capsules. However, such unused genomic material can still be the source of valuable agricultural features.

To obtain the genome of the convar. *crepitans*, we performed DNA sequencing on the Oxford Nanopore Technologies and Illumina platforms. We assembled the received data using a range of software and calculated quality statistics. Different assemblers were tested in our previous work on their efficacy in constructing the genome of *L. usitatissimum* line 3896 (Dvorianinova et al., 2022). Most of the tested software (miniasm, NextDenovo, Raven, Shasta, SMARTdenovo, and wtdbg2) demonstrated poor QUAST and BUSCO statistics or assembled a genome of a significantly smaller size than the expected one. In our work on sequencing the genome of the Atlant cultivar, two tested tools (Shasta and wtdbg2) also showed poor performance (Dmitriev et al., 2020b). Therefore, we decided not to include the aforementioned assemblers in our current analysis and focused on the recently released tools and those that showed the best results.

Thus, to obtain draft assemblies, we used Canu, Flye, NECAT, and GoldRush. Canu, the most CPU time-consuming tool, still demonstrated the best performance in terms of assembly completeness and contiguity, including assembly size. NECAT produced the assembly with the highest N50 and the fewest number of contigs but of a size smaller than the expected one (400–450 Mb) and ∼42 Mb smaller than that for the assembly by Canu. Both assemblies had the same BUSCO completeness. Flye assembled a genome with QUAST and BUSCO statistics that was significantly worse but comparable to those of the assemblies by Canu and NECAT. At the same time, the assembly by Flye had the smallest relative number of mismatches/indels. Possibly, this could be due to the included polishing module (Kolmogorov et al., 2019). However, despite the achieved accuracy, the whole genome sequence still missed 20%–30% of the expected genome size. GoldRush was unable to produce a genome with reasonable statistics. Thus, we considered the assembly by Canu optimal.

**FIGURE 3:**
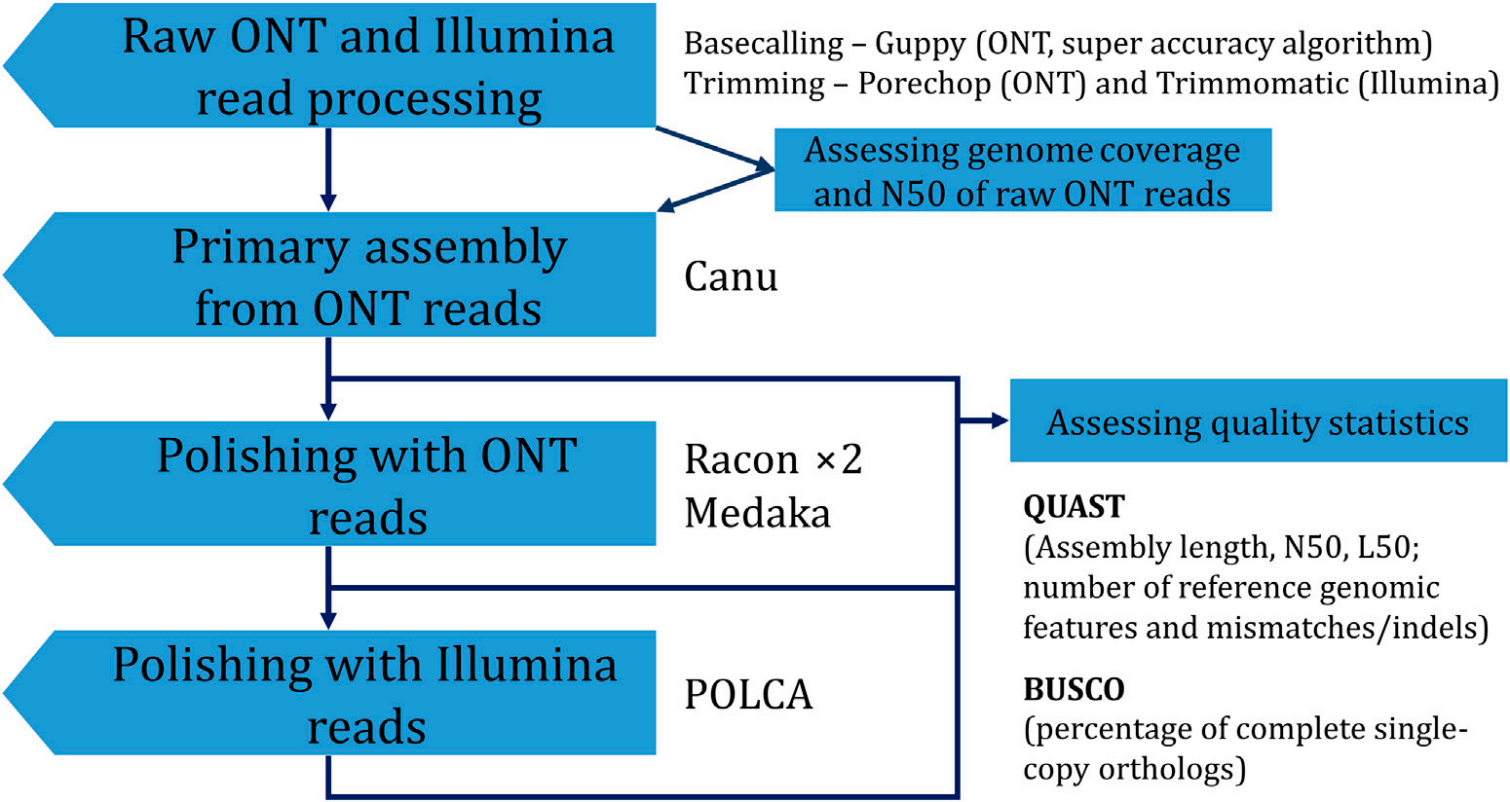
The scheme used to obtain the final genome assembly of *L. usitatissimum* convar. *crepitans*.

To improve the accuracy of the obtained genome assembly, one can apply a polishing procedure. The Canu-assembled genome was polished using ONT reads by the Racon (two iterations, both R.9.4.1 and R10.4.1 reads) and medaka (R10.4.1 reads) polishers. Each of the two rounds of Racon increased BUSCO completeness and the number of complete reference genomic features in the assembly. The procedure also decreased the relative number of mismatches and indels by 12.5% and 46.1%, respectively. Sequencing data from R9.4.1 flow cells are more inaccurate than those from R10.4.1 flow cells (Sereika et al., 2022). Thus, in our previous study, polishing with ONT data only from R9.4.1 flow cells had a less dramatic effect (Dvorianinova et al., 2022). Polishing with medaka showed the same trend in statistic values as polishing with Racon. Final polishing with Illumina reads by POLCA also improved QUAST and BUSCO parameters. However, skipping polishing with ONT reads and polishing only with Illumina reads was not as beneficial as using both ONT and Illumina data. BUSCO completeness was almost the same for assemblies obtained according to Canu—POLCA and Canu—Racon ×2—medaka—POLCA. However, more mismatches/indels remained in the assembly polished only with Illumina reads. Thus, the final optimal assembly was obtained using the Canu—Racon ×2—medaka—POLCA scheme (Figure 3). The assembly had a size of 412.6 Mb, consisted of 1,483 contigs, had an N50 of 5.2 Mb, and a BUSCO completeness of 94.6%.

BUSCO completeness of the obtained assembly was higher than that of the available assemblies for *L. usitatissimum*. Its length and repeat content were expectedly greater than these parameters of the assemblies obtained from short reads (varieties CDC Bethune, Longya 10, Heiya 14; *L. bienne* 15003). However, the repeat content in the genome of the convar. *crepitans* was similar to that of the assemblies from long reads (varieties 3896, Atlant, Neiya No. 9, and YY5 v.2). Thus, the non-coding sequences in the assembly are likely complete*.* The percentage of duplicated BUSCO in the obtained assembly was also high (above 60%) and comparable to that of *L. usitatissimum* assemblies. This fact correlates with the idea of *L. usitatissimum* origin. The species might have originated from the crossing of two *Linum* species. Then, the genome of the progeny probably underwent diploidization. Thus, the resulting ploidy of most genomic features is four (Bolsheva et al., 2017).

The assembled genome of the convar. *crepitans* has a quality comparable to that of the line 3896—the NCBI reference genome for *L. usitatissimum*. The line 3896 genome was assembled and polished using ONT reads (Dvorianinova et al., 2022). Meanwhile, the genome of the convar. *crepitans* was assembled from ONT data and additionally polished with both ONT and Illumina reads. Thus, the assembly of the convar. *crepitans* has more complete BUSCO likely due to the improvement with accurate Illumina data. However, its contig N50 is lower than that of the assembly of line 3896 or variety YY5. In general, the obtained genome of the convar. *crepitans* has a quality close to that of most flax assemblies from long reads and outperformed the assemblies from short reads. Nevertheless, its level can still be upgraded to the chromosome one, e.g., using Hi-C data.

In this work, we sequenced the first genome of *L. usitatissimum* convar. *crepitans*. The volume and quality of the obtained data were sufficient to produce a high-quality assembly with QUAST and BUSCO statistics that were superior or close to those of the available *L. usitatissimum* genomes. Its quality level can be additionally upgraded to the scaffold and chromosome level. Our data allow investigating the diversity and evolution of the section *Linum* as well as mining key traits for breeding.

## Data Availability

The datasets presented in this study can be found in online repositories. The names of the repository/repositories and accession number(s) can be found as follows: https://www.ncbi.nlm.nih.gov/, PRJNA1006423.
